# Optimizing total ammonia–nitrogen concentration for enhanced microbial fuel cell performance in landfill leachate treatment: a bibliometric analysis and future directions

**DOI:** 10.1007/s11356-023-28580-z

**Published:** 2023-07-15

**Authors:** Aliyu Ishaq, Mohd Ismid Mohd Said, Shamila Binti Azman, Mohd Firdaus Abdulwahab, Zainab Toyin Jagun

**Affiliations:** 1 Department of Water and Environmental Engineering, School of Civil Engineering, Faculty of Engineering, Universiti Teknologi Malaysia, 81300 Johr Bohr, Malaysia; 2 Department of Water Resources and Environmental Engineering, Ahmadu Bello University, Kaduna, 1045 Zaria Nigeria; 3 Department of Biosciences, Faculty of Sciences, Universiti Teknologi Malaysia, Johor Bahru, Malaysia; 4 Department of Real Estate, School of Built Environment Engineering and Computing, Leeds Beckett University, City Campus, Leeds, UK

**Keywords:** Ammonia inhibition, Bibliometric analysis, Landfill leachate, Microbial fuel cells (MFCs), Sustainable energy

## Abstract

Untreated landfill leachate can harm the environment and human health due to its organic debris, heavy metals, and nitrogen molecules like ammonia. Microbial fuel cells (MFCs) have emerged as a promising technology for treating landfill leachate and generating energy. However, high concentrations of total ammonia–nitrogen (TAN), which includes both ammonia and the ammonium ion, can impede MFC performance. Therefore, maintaining an adequate TAN concentration is crucial, as both excess and insufficient levels can reduce power generation. To evaluate the worldwide research on MFCs using landfill leachate as a substrate, bibliometric analysis was conducted to assess publication output, author-country co-authorship, and author keyword co-occurrence. Scopus and Web of Science retrieved 98 journal articles on this topic during 2011–2022; 18 were specifically evaluated and analysed for MFC ammonia inhibition. The results showed that research on MFC using landfill leachate as a substrate began in 2011, and the number of related papers has consistently increased every 2 years, totaling 4060 references. China, India, and the USA accounted for approximately 60% of all global publications, while the remaining 40% was contributed by 70 other countries/territories. Chongqing University emerged as one of the top contributors among this subject’s ten most productive universities. Most studies found that maintaining TAN concentrations in the 400–800 mg L^−1^ in MFC operation produced good power density, pollution elimination, and microbial acclimatization. However, the database has few articles on MFC and landfill leachate; MFC ammonia inhibition remains the main factor impacting system performance. This bibliographic analysis provides excellent references and future research directions, highlighting the current limitations of MFC research in this area.

## Introduction

Due to its high concentrations of toxins and pollutants, particularly heavy metals and ammonia, landfilling leachate presents a substantial issue in managing municipal solid waste (MSW) (Li et al. 2020; Mahmoudi et al. 2020). One discharge that threatens the ecosystem, landfill leachate, calls for immediate attention, particularly in developing nations where open dumping is common. The type of MSW, location, and stage of landfill degradation all affect the composition and characteristics of leachate (Miao et al. 2019). The treatment of leachate is complicated by high-molecular-weight organic molecules, hydrocarbons, and inorganic salts (Xia et al. 2018). In MSW, leachate treatment techniques must be efficient and sustainable (Liu et al. 2021).

Direct dumping of untreated leachate into neighbouring water bodies pollutes the surface and groundwater, putting the health of the surrounding ecosystem, soil, and people at risk (Rahmani et al. 2022; Jagun et al. 2022). Leachate treatment is essential. Leachate is complicated and challenging to manage cost-effectively. The plan should increase nitrogen removal while reducing operating expenses (Zhang et al. 2019). Although physical–chemical treatments eliminate many pollutants, they are expensive and produce additional pollutants (Li and Chen 2018). For example, biological therapies that remove ammonia are less expensive and produce no secondary pollutants (Kamaruddin et al. 2017).

Moreover, research has demonstrated that biological treatment is a more economical solution to address several environmental water issues (Zhu et al. 2021). Microbial fuel cells (MFC) have recently been more popular as a possible technique for energy production and wastewater treatment (Santoro et al. 2019). Microbial fuel cells (MFCs) have advanced in design, operating dynamics, combinations, and testing thanks to the groundbreaking work of Bruce E. Logan’s group at Pennsylvania State University in the USA (Logan 2010).

MFCs are a promising technique for treating landfill leachate because they can efficiently remove contaminants while simultaneously generating energy. MFCs operate by harnessing the metabolic activities of microorganisms to generate a voltage between two electrodes. Organic matter serves as a fuel source for the bacteria, oxidizing the organic matter and producing electrons as a by-product (Brown et al. 2015). MFCs have been successfully used in several studies to remediate landfill leachate, including the removal of ammonia–nitrogen (${\mathrm{NH}}_{4}^{+}$-N), total nitrogen, and chemical oxygen demand (COD) (Hassan et al. 2018a, b; Zhang et al. 2015). Power densities of 5.5 to 15.1 W/m^3^ have been observed for MFCs, indicating their efficiency in producing electrical energy from landfill leachate. (Hassan et al. 2018a, b; Hussein et al. 2021; Jiang et al. 2019; Yaashikaa et al. 2022). Ammonium (${\mathrm{NH}}_{4}^{+}$) is the dominant species when dissolved in the aqueous phase; the generation of ${\mathrm{NH}}_{3}$ is influenced by total ammonia nitrogen (TAN) concentration, pH, and temperature. ${\mathrm{NH}}_{3}$ can act as a proton acceptor, reducing the availability of protons in the anode compartment, which limits the production of electrons. Furthermore, ${\mathrm{NH}}_{3}$ can interfere with the electron transfer processes within the microbial populations, reducing the current generation rate (Deng et al. 2021).

Researchers have endeavoured to write review papers on MFC’s efficiency, research gaps, and recent advances in leachate treatment, but they have yet to provide any information on overall research trends, which could be determined through bibliometric analysis on MFC and landfill leachate. The most similar evaluation to this study only examined the overall trend of MFC and landfill leachate without considering bibliometric analysis in conjunction with reviewing the factors influencing the performance of MFC, like TAN inhibition. A bibliometric analysis that utilizes mathematical, statistical, and graphical tools to analyse publications and identify research gaps is necessary to comprehend a study area’s current state and development trends (Ilmasari et al. 2022; Liu et al. 2021). Descriptive statistics, such as the geographic distribution of publications and the most popular search terms, and citation analysis, which is the assessment of partnerships between authors, journals, and nations and is primarily used to identify and illustrate research development patterns, are the two main components of bibliometric analyses (Diodato and Gellatly 2013; Xue et al. 2021). Additionally, this review paper discovered a dearth of information on the topic of “MFC and landfill leachate” accessible from the two databases, especially studies on ammonia inhibition generally. This emphasizes the value of conducting more research in this field. Several methods have countered the effects of ammonia–nitrogen inhibition.

## Methods

For this systematic search, two databases—Scopus and Web of Science—were used, and the search terms used were “microbial fuel cells” and “landfill leachate.” The study is based only on original research articles, review papers, and conference papers. To maintain the quality of the review, all duplicates were identified and merged to provide consistency across the review. Each research piece was after that subjected to a thorough evaluation. This review article used two approaches: bibliometric analysis and the standard review method.

### Bibliometric and standard review methods

Bibliometric analysis is a quantitative method that analyses scholarly publications’ bibliographic data. This method can provide insight into the impact and popularity of research papers and identify important authors and journals in a specific field. On the other hand, a standard literature review is a qualitative method that involves critically analysing and synthesizing the findings from a collection of research papers to conclude the current state of knowledge on a specific topic (Chen et al. 2022). Combined, these methods permit quantitative and qualitative examination of the relevant literature, leading to a richer understanding of the subject. For instance, a bibliometric analysis can identify the most cited articles, authors, and journals related to MFC (microbial fuel cell) and landfill leachate, while a standard literature review can provide a qualitative analysis of the content of those papers. Combining both approaches can provide a comprehensive understanding of a topic, allowing for the identification of important research and trends, as well as gaps and limitations in the literature (Aria and Cuccurullo 2017). This integrated approach can inform future research directions and policy decisions). Two scholarly publishing databases are used in bibliometric studies to evaluate the most recent state-of-the-art research on a particular subject. This review aims to evaluate both strategies critically. The method is broken down into three steps: (i) database searches on the internet, (ii) article screening, and (iii) final statistics and refining. Data were extracted for this research from the Scopus and Web of Science databases (Burnham et al. 2006). A bibliometric analysis study is a mechanical method to understand the global research trends in a particular field based on the findings of the academic literature database.

### Source of data and search approach

**Fig. 1:**
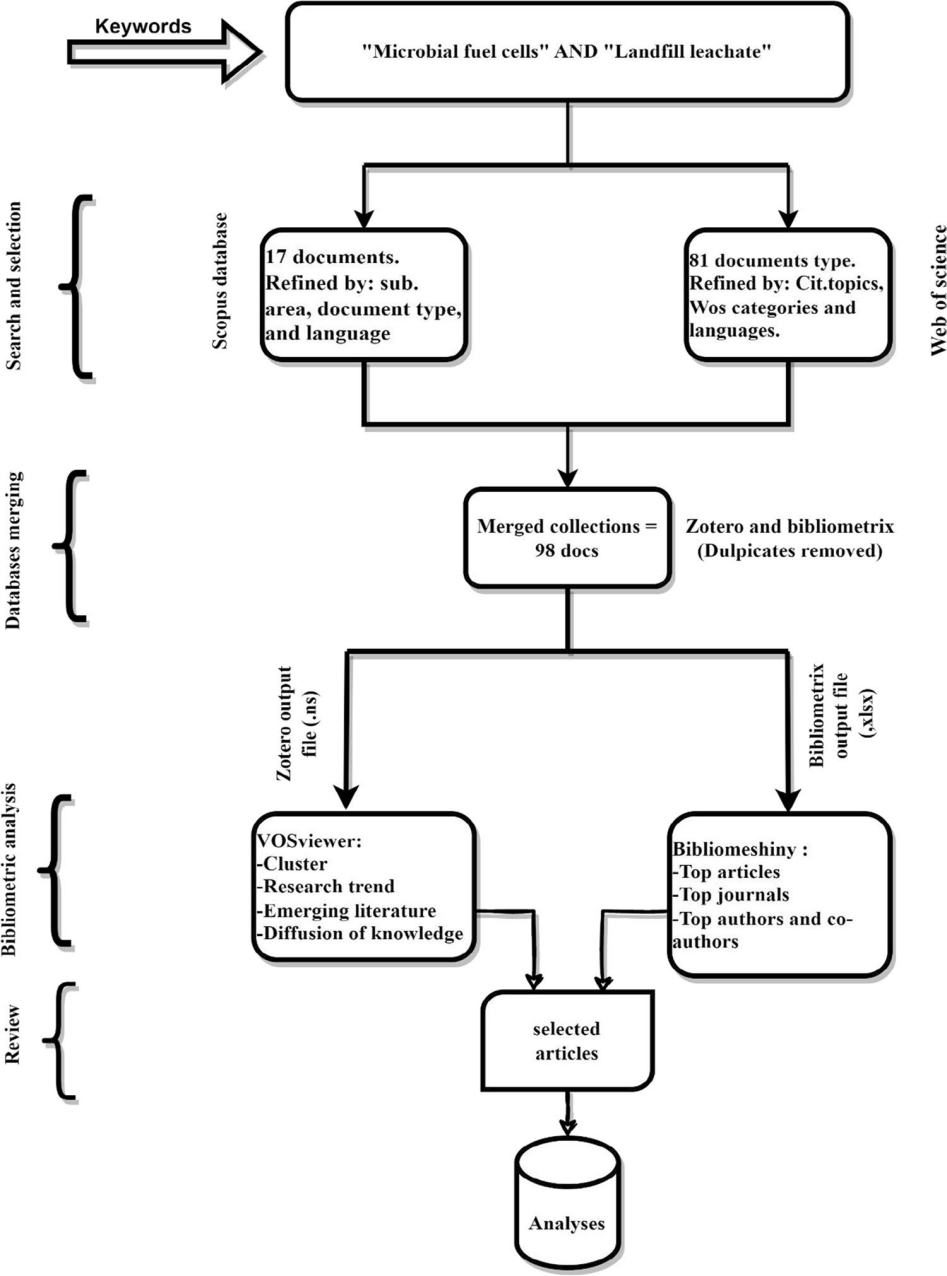
Flowchart of gathering data of publications for review analysis

On February 8, 2023, bibliometric statistics were retrieved from Scopus and Web of Science online databases. To find articles published between 2006 and 2023, the keyword search string: "Microbial fuel cell" AND "Landfill leachate*" was used. Apart from journals and articles, all document types were dropped, leaving 98 documents, 17 for Scopus, and 81 for Web of Science. To fully examine research trends, this study only looked at research articles. The search results were then exported in RIS format, complete with reference, bibliographic, abstract, and keyword data, for bibliometric analysis. as shown in Fig. 1.

### Data analysis

To identify research trends, popular subject areas, and the nations, affiliations, authors, and journals with the most publications, the data gathered from a search query underwent a variety of analytics. The documents that resulted were archived in RIS format. After merging and eliminating duplicates with the Zotero software, the papers were uploaded into VOSviewer, version 1.6.18 (Leiden University, Leiden, Netherlands), to produce bibliometric maps displaying co-authorship and keyword co-occurrence. The size and spacing of the circles on these maps, which are made up of circles connected by network linkages, indicate word importance and reliance. A greater link strength rating indicates a stronger connection between the two terms. The information collected from the search string was subjected to various analyses, including research patterns, the most popular subject areas, and the most active nations (Ilmasari et al. 2022).

In this study of keyword co-occurrences, the minimum keyword occurrence was set at two (2), and 123 out of 501 keywords met this threshold. Synonyms were consolidated to streamline related keywords before loading the data into VOSviewer. For example, terms like “leachates,” “landfill leachate,” “fresh leachate,” and “ancient leachate” were combined as “leachate.” “Fuel cell” was also synonymous with “microbial fuel cells.” The VOSviewer network visualization mode displayed a map based on the average publication year of the keywords. The strength of keyword co-link occurrence and citation network reinforced this representation. In the co-authorship analysis, 386 authors from different countries were considered, with only 79 meeting the threshold. The study examined co-author relationships across America, Asia, and Europe, categorized by affiliated countries and territories.

## Results and discussions

### Publication output and growth of research interest

**Fig. 2:**
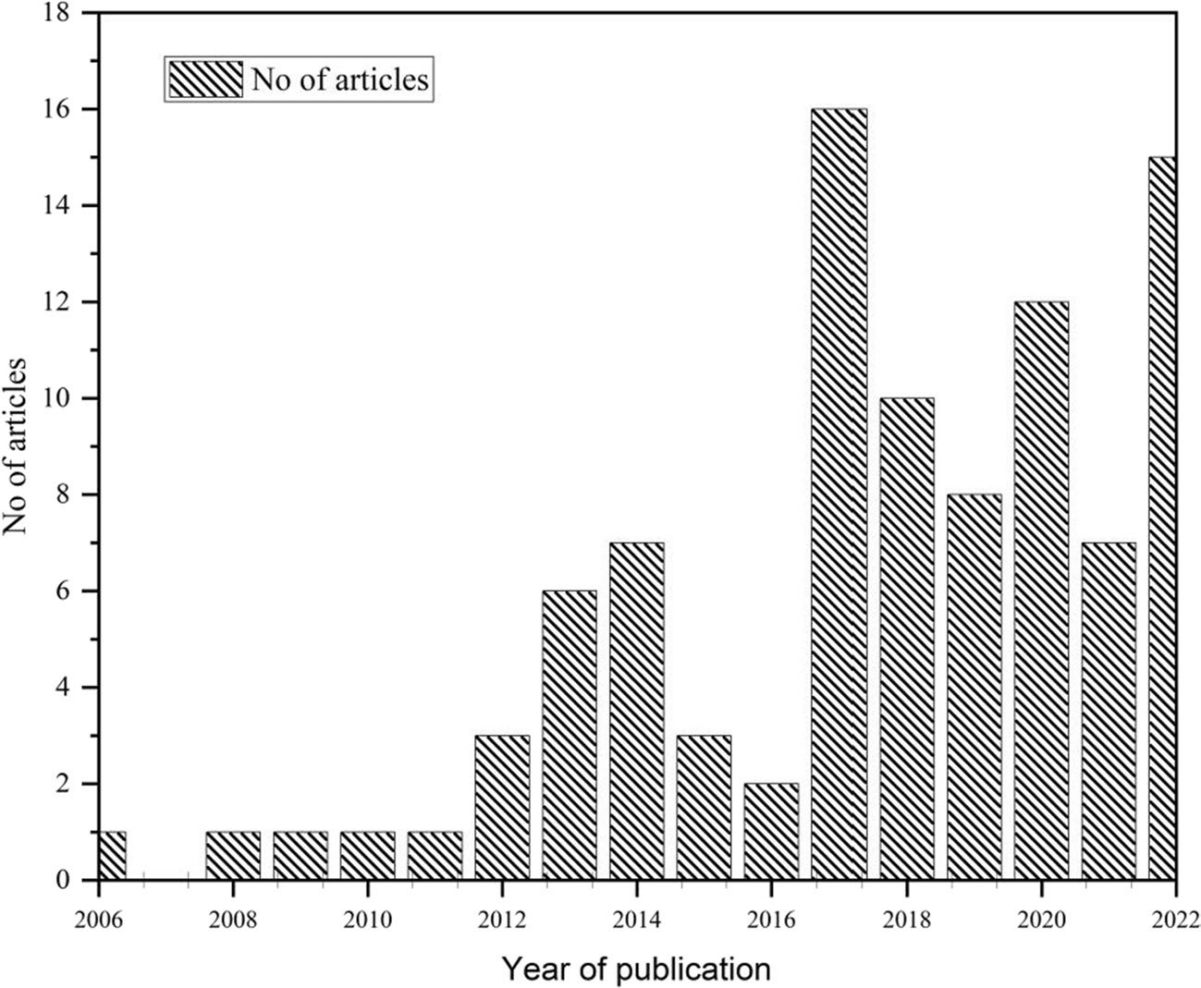
The annual research articles indexed in Scopus and web of science from 2006 until 2023

For the past 17 years, 98 scholarly articles have been published (as shown in Fig. 2). Research on MFCs has been ongoing since 1962 (Murugesu et al. 2022). However, using landfill leachate as a substrate did not start until 2006. The average yearly growth rate (AGR) increased until 2012 when the output of scholarly articles nearly doubled. The overall number of published articles has increased greatly due to the ongoing growth in annual publishing output. It is expected that the rising rate of annual publications will continue. However, it is noted that some articles are open access while others have restricted access. It has been noted that articles published in open-access journals are more likely to receive more citations. The subject of MFC research is large, and numerous research groups worldwide are actively working in this area. According to a topic area analysis, environmental challenges are the primary focus of MFC investigations, as evidenced by the inclusion of most relevant keywords such as landfill leachate (58), energy generation (31), removal (21), performance (20), wastewater treatment (17), MFC (26), and ammonia–nitrogen (35).

### Preferred journals

Table 1 offers details of 10 scientific journals, including their serial number, name, the total number of publications (TP), the total number of citations received globally (TC), CiteScore for 2022, publisher, impact factor for 2021, quartiles of the journal, and *H*-index. The CiteScore metric assesses a journal’s effect based on the typical number of citations per document over 3 years (Khudzari et al., 2018). The average number of citations earned by articles in a journal over the past 2 years is used to calculate the impact factor. The quartiles show the journal’s ranking in relation to other journals in the same field based on its impact factor. The top 25% are represented by Q1, the next 25% by Q2, and so forth. Based on the number of publications and the number of citations such articles have earned, the *H*-index is a metric that assesses both the productivity and influence of a scientist’s or scholar’s published work.

**Table 1:** The top 10 most productive journals on MFC and landfill leachate research with their most cited article

S/N	Journal source	Total publication (TP)	Global citations (GC)	CiteScore 2022	Publisher	Impact factor	Quartiles	*H*-index (Scimago)
1	International Journal of Hydrogen energy	6	144,512	11	Elsevier	7.139	Q1	231
2	Chemosphere	4	177,874	13.1	Elsevier	8.943	Q1	265
3	Journal of Applied Biology and Biotechnology	3	691	1.5	Open Science Publishers LLP	0.93	Q3	8
4	Environmental science and pollution	3	117,496	14.7	Springer	5.190	Q2	132
5	Environmental technology	3	23,452	5.4	Taylor & Francis	3.475	Q1	78
6	Environmental Chemical Engineering	3	56,387	9.3	Elsevier	7.968	Q1	90
7	Journal of hazardous materials	3	188,186	19.9	Elsevier	14.224	Q1	307
8	Science of the total environment	3	471,465	16.1	Elsevier	10.753	Q1	275
9	Chemical Engineering Journal	2	372,656	21.3	Elsevier	16.744	Q1	248
10	Electrochimica Acta	2	89,870	12.7	Elsevier	7.336	Q1	249

The studies revealed that the top ten most productive journals are held by two unique publishers (Table 1). Elsevier is the company that publishes most of the top ten journals in this area. The most productive journal was the International Journal of Hydrogen Energy, which accounted for nearly 20% of all publications with 6 papers, followed by the Journal of Chemosphere (12.3%) with 4 articles. According to the CiteScore 2022 report, seven journals had CiteScores of 7 or above. The CiteScores for Chemical Engineering Journal (21.3) and Journal of Applied Biology and Biotechnology (1.0) was the highest and lowest, respectively. The total number of citations and the CiteScore of the most prolific journals were much higher. This was most likely because English is the most used language for publication, and many journals are classed Q1 with an impact factor greater than 5.1. Even though Environmental Technology and the Journal of Applied Biology and Biotechnology had the lowest impact factors, at 0.93 and 3.43, respectively. Science of the Whole Environment has received the most global citations, totalling 471,465. Elsevier publishes this journal, which has an impact factor of 10.753, which is considered high. It is classified as a Q1 journal, which means it is in the top 25% of journals in its field. The journal publishes original research and review articles and shows that the research published in the journal is significant, and its findings have been frequently used and cited by other academics, reflecting the impact, prestige, and influence of the research published within the Journal.

On the other hand, the Journal of Applied Biology and Biotechnology has the fewest global citations, with only 691. Open Science Publishers LLP publishes this journal with a low impact factor of 0.93. It is classified as a Q3 journal, which means it is among the bottom 25% of journals in its field. This journal has the advantage of being open-access and publishing original research, review articles, and case studies in applied biology and biotechnology. This could be due to the quality of the study, the journal’s visibility and accessibility, or a lack of promotion or marketing activities.

Additionally, it was established that CiteScore might hold weight in certain authors’ minds when deciding which journals are suitable for publishing their latest and most important research. However, CiteScore should not be the sole determinant of a journal’s value. Rather, authors can consider whether the journal can deliver their work to the right audience and positively contribute to advancing the field. In other words, the relevance and impact of a journal on the specific research topic should also be considered alongside the CiteScore. This way, authors can make more informed decisions about where to publish their work (Chen et al. 2020; Ilmasari et al. 2022).

### Leading countries, top institutions, and international collaboration

**Fig. 3:**
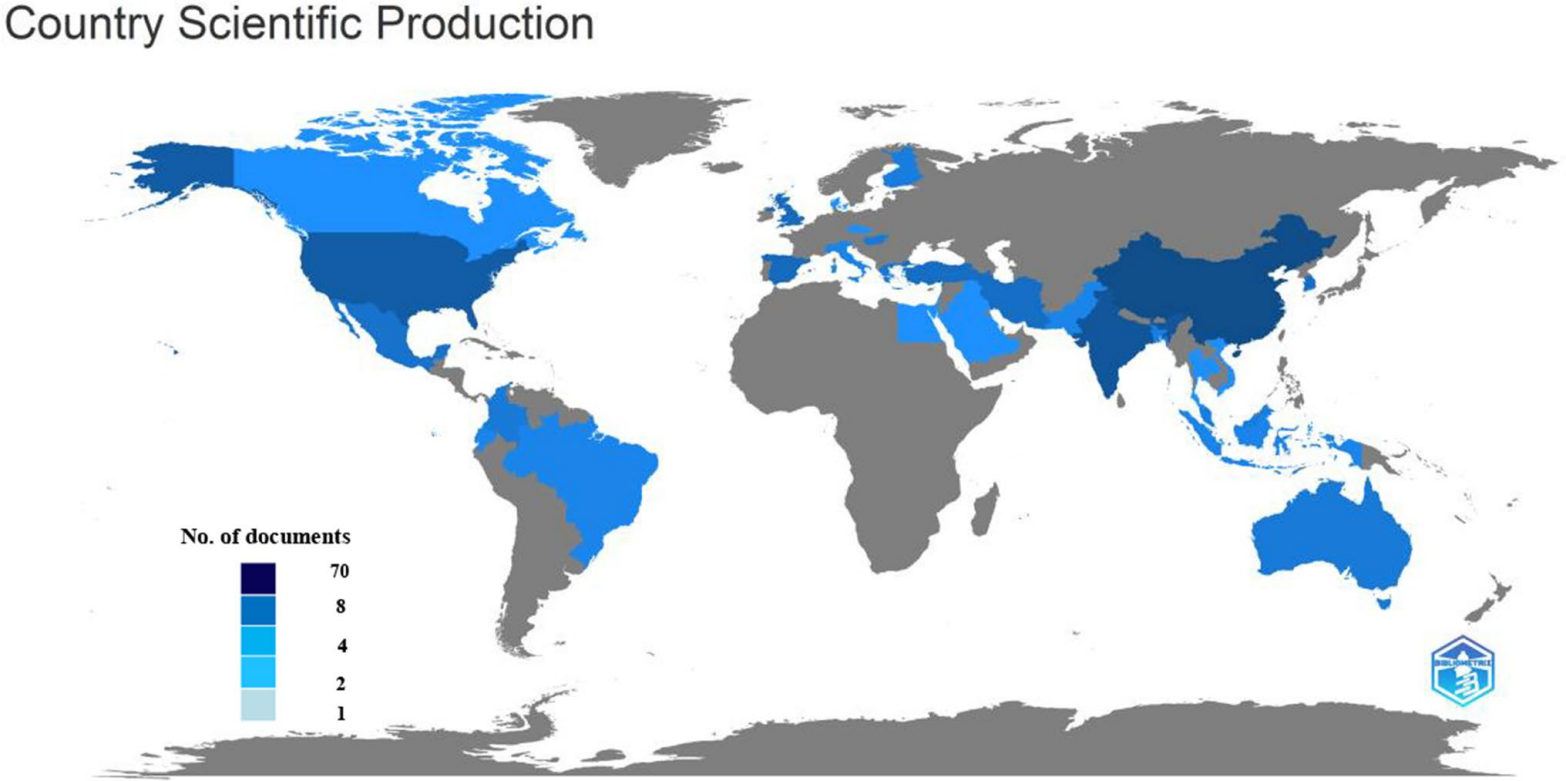
Geographical distribution and their affiliations in MFC and landfill leachate publications

**Fig. 4:**
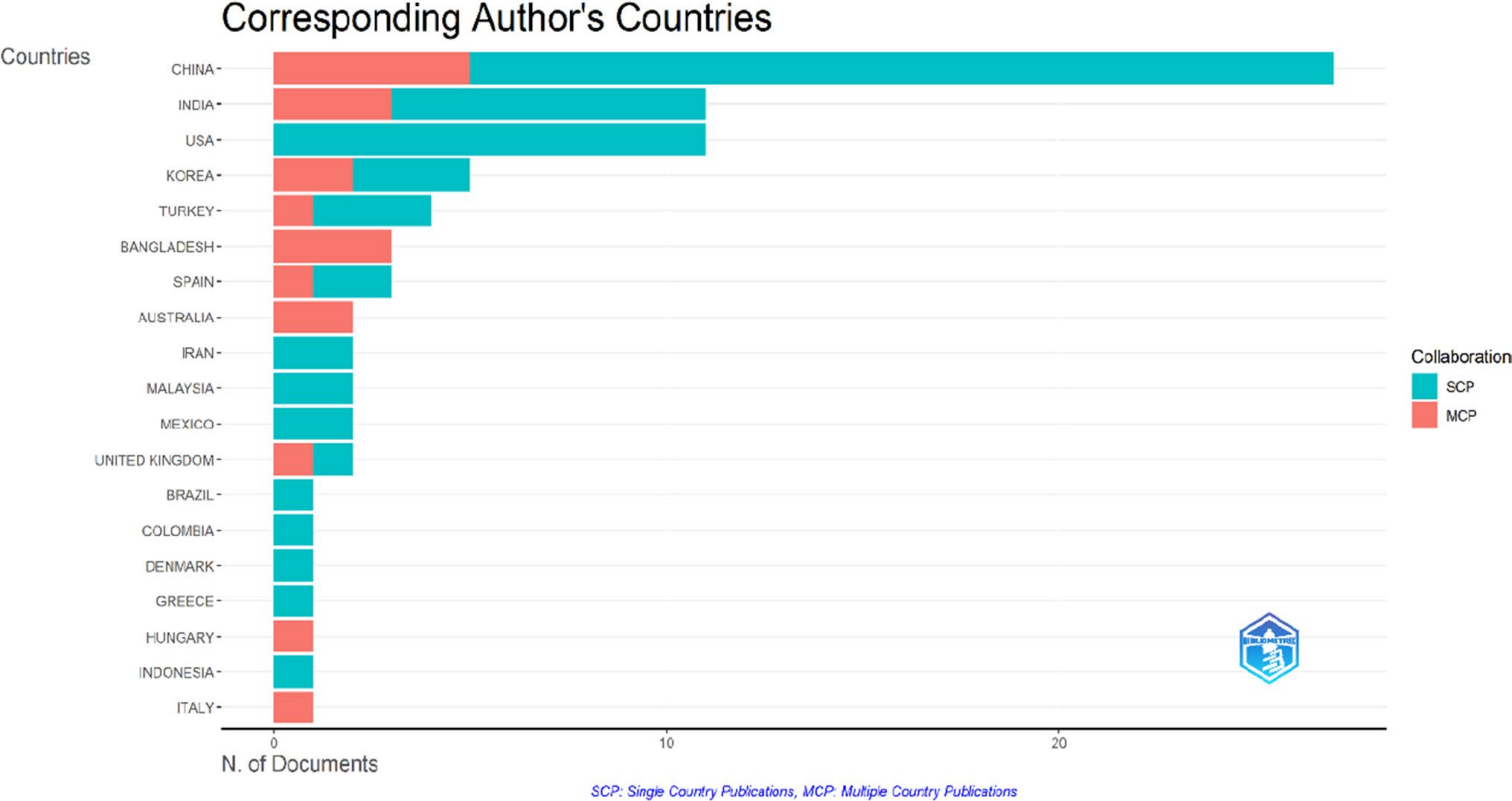
Corresponding author’s countries and collaborations in MFC and leachate publication

The top 5 most productive nations in the world, according to Fig. 3, are involved in more research on MFC and landfill leachate as a substrate. China, India, and the USA made up almost 60% of all international papers, highlighting their importance in advancing this area of research. China was the most productive country, with 70 articles in 98 journals; India and the USA ranked second and third, with 8 and 4 articles, respectively, as described in Fig. 4.

A diverse group of institutions has conducted MFC and landfill leachate research. According to online data from Scopus and Web of Science, 98 institutions have contributed to the publication of research articles on this topic, including both academic and non-academic institutions such as research centre, etc. The top 5 most productive academic institutions in this research area are Chongqing University (7 articles), Indian University (5 articles), Girona University (5 articles), West England University (5 articles), and Virginia Polytechnic (5 articles). Chongqing University in China was the most productive institution, having published seven articles on this topic. Indian universities in India and West England universities in the UK were the next most productive institutions. This suggests that significant research is being conducted on this topic in China, India, and the USA and that these countries are major contributors to the global research output on MFC and landfill leachate. Overall, this provides insights into the factors that may contribute to the distribution of research output across institutions and countries, as well as the research landscape on MFC and landfill leachate.

The fact that multiple institutions from different countries have contributed to the research on MFC and landfill leachate suggests that this topic is of interest and importance to researchers worldwide. The diversity of institutions also indicates that various disciplines, including engineering, environmental science, and microbiology, are researching this topic. The fact that the top productive academic institutions are in different countries suggests significant collaboration among researchers working on this topic in different parts of the world, as seen in Fig. 4. This shows that there is a significant intra-country collaboration between these countries. In contrast, Indonesia had the lowest percentage of SCP at 7.3%. Sixty-two out of 98 publications linked to numerous affiliations from multiple countries. International collaboration has many benefits, including a wider network, knowledge sharing and experience exchange, and an effective strategy for ranking up. Moreover, it is noteworthy that some of the universities listed in the publications are ranked among the top 100 universities in the world, indicating that the MFC field has garnered significant attention and recognition from leading academic institutions worldwide. This is a testament to MFC technology’s growing importance and potential in the global scientific community and its potential for real-world applications in various fields.

While some countries have many publications on MFC and landfill leachate, few of their institutions are ranked among the top 10 most prolific academic institutions on this topic. This shows that research in these countries is not centralized, which could be a possible reason. Research published in languages other than English may be excluded from international databases and not counted in the analysis. Therefore, the research output from these countries may not be fully captured in databases like Scopus and Web of Science, which could lead to an underestimation of the research output of these countries.

### Analysis of keyword occurrence and country co-authorship and research collaborations

**Fig. 5:**
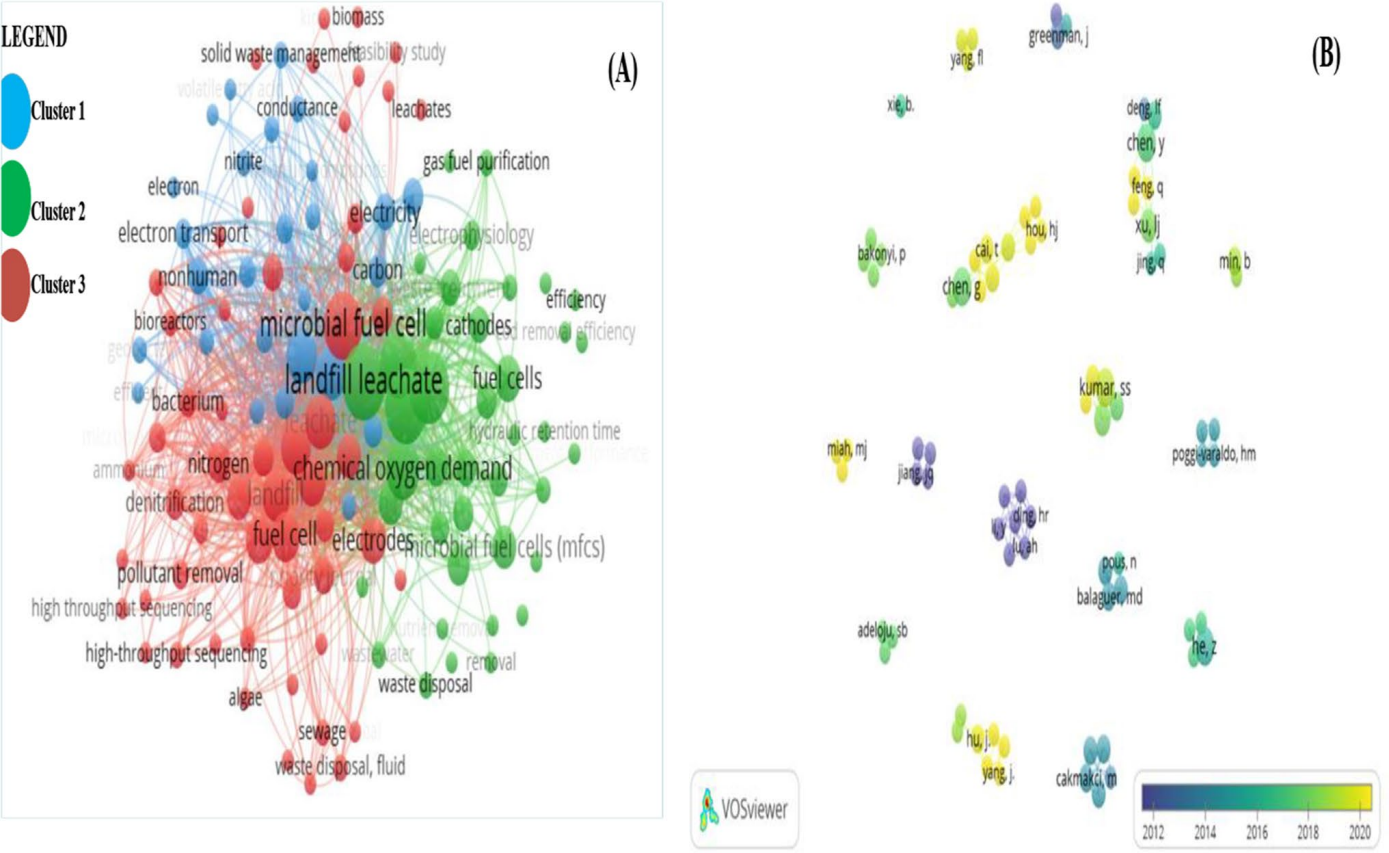
**A** and **B** A screenshot of the three distinct clusters of the bibliometric map produced using the network visualization mode and the co-occurrence of author keywords, respectively. The performance of bioelectricity generation is most closely correlated with the blue cluster, followed by the green cluster, the mechanism research, and the maroon cluster, the elimination of refractory pollutants. Figure B shows the visualization of co-authorship overlaps in MFC and landfill leachate studies from 2006 to 2022 is shown on a map

Analysing key terms using the VOSviewer software is useful to understand better the research areas and tendencies in a specific field. In the case of MFC and landfill leachate, the generated term co-occurrence network map helps identify the main research clusters. As shown in Fig. 5A, the terms are divided into three clusters, represented by different colours: blue, green, and red. The circles’ size reflects the term’s frequency, while the node’s colour indicates its cluster. The blue group contains keywords such as “MFC,” “solid waste management,” “nitrite,” and “electron transfer” and is primarily concerned with the efficiency of bioelectricity generation. This grouping indicates that studies are being conducted to enhance the effectiveness of MFCs in producing bioelectricity and dealing with solid waste. The cathodes, fuel cells, HRT, and microbial community are all examples of concepts in the green cluster, which focuses on investigating the underlying mechanisms. This concentration of studies provides evidence that scientists are actively investigating the relationship between MFCs and landfill leachate remediation. Refractory pollutant removal (shown by the maroon cluster) is a significant use case for MFC and landfill leachate treatment. Nitrogen removal, sequencing, total nitrogen, ammonia, denitrification, pollutant removal, and carbon source are all related concepts. This grouping of studies implies that efforts are being made to enhance MFC technology’s ability to remove conventional pollutants from landfill leachate. Overall, the trends and directions of current MFCs and landfill leachate treatment research are illuminated by analysing important phrases using the VOSviewer software. To better grasp the present state of knowledge and identify research gaps and possibilities, researchers and practitioners can use the discovered clusters to highlight the important study components in this field.

Following the major authors and research groups in a field is the best way to keep up with the latest developments. It is also used to determine the most effective authors and teams and how they work together. The authors’ ability to work together improves with the size of the cycle of co-authorship. This is because authors who have worked together for a long time and have established a good working relationship will have a high cycle of co-authorship. It 'is possible that they have worked together before, have similar research interests, and possess complimentary abilities.

Figure 5B displays the results of a bibliometric study that determined which research groups had the most impact on the development of MFC and landfill leachate. Figure 5B shows the large and small collaboration groups of authors working; it is possible to highlight the greater collaboration network that includes clusters in the blue, green, and maroon colours with 28 authors. Scientific research collaboration is essential for advancing knowledge and understanding in a particular field. Researchers can share their expertise and resources to achieve a common goal through collaboration. Collaborating with a diverse range of researchers can bring fresh perspectives and ideas, leading to breakthroughs and discoveries. The diagram shows that Hou J has collaborated with several other researchers, with Miah M. J. the most frequent collaborator. A larger number of collaborations between Hou J. and Miah M. J. could suggest a close working relationship between the two researchers. They may share common research interests or work together on specific projects. The other researchers listed, such as Kumar H., Yang J., Cai T., Feng G., Min B., and Kumar S. S., may also have collaborated with Hou J. on some level but to a lesser extent. In addition, Hou J. and Miah M. J. have a significant working relationship, which may have implications for their research and future developments in their field.

### The top 10 most productive authors with local and global citations

Table 2 ranks 10 authors according to their local and global citation counts, *H*-index, and overall link strength. These measures are frequently used to evaluate research impact and productivity. Balaguer, Colprim, and Puig, the first three authors, had the greatest worldwide citation counts, ranging from 4630 to 5262. This shows that their study has received many citations and has influenced their respective disciplines. Furthermore, all of them have *H*-indices of 43 or above, indicating that they have produced many publications referenced by other scholars. Their overall link strength of 8 indicates that they collaborate closely with other authors and that their work is well incorporated into the larger research community. The remaining seven authors have fewer global citation counts and *H*-indices, indicating that their research impact may be more limited. However, they all have local citation counts of 31 or above, indicating that their work is well-regarded within their own institution or research community. The total link strength values for these authors are also relatively high, suggesting that they have strong connections with other researchers and institutions; of note is the fact that Cabré, Coma, and Serra have low *H*-indices and total link strength, which suggests that their research impact may be more limited. However, they have local citation counts that are relatively high, indicating that their work is still respected and valued within their institution or research community.

**Table 2:** The top 10 most productive authors with local and global citations

S/N	Author	Local citation	Global citation	*H*-index	Total link strength
1	Balaguer, M. D	44	4969	43	8
2	Colprim, Jesús	44	5262	45	8
3	Puig, Sebastià	44	4630	43	8
4	Cabré, Marina	36	384	3	7
5	Coma, Marta	36	1745	22	7
6	Serra, Marc	36	429	4	7
7	Jiang, Junqiu	31	2140	25	6
8	You, Shijie	31	5312	44	6
9	Zhang, Jinna	31	1490	17	6
10	Zhao, Qiangliang	31	7025	47	6

Overall, these results suggest that the top three authors have the strongest research impact and productivity, while the remaining authors may have a more limited impact but are still respected within their research community. Total connection strength values also imply that researchers’ ability to collaborate and integrate significantly influences the impact and productivity of their work.

### Analysis and interpretation of the selected articles

Both bibliometric analysis and traditional review approaches, addressed in detail throughout this article, contribute to a more in-depth grasp of the relevant literature on this topic. The most prominent studies and authors on a topic can be found through bibliometric analysis, while a traditional literature review can help to discover the most important themes and trends in the literature. This review combines these methods to pinpoint unexplored questions, interesting avenues for further study, and existing knowledge gaps. This literature study seeks to understand better the parameters that influence the efficiency of microbial fuel cells (MFCs), focusing on the effect of ammonia–nitrogen from landfill leachate. Eighteen of the ninety-eight papers relevant to MFCs and landfill leachate discussed the impact of ammonia inhibition in MFCs. The 18 selected articles were analysed, as shown in Table 3. This information is valuable for researchers working in this field as it provides insights into the optimal conditions for MFC performance in the presence of ammonia.

**Table 3:** Previous studies of ammonia–nitrogen inhibition on MFC performance

S/N	Influent (mg L^−1^)	Reactor type (MFC)	Inoculation	Mode	°C	pH	Period (days)	Inhibiting conc.(mg L^−1^)	Optimum conc. (mg L^−1^)	Removal efficiency (%)	Minimum PD (mWm^−3^)	Optimum PD (mW m^−3^)	Reference
1	84–4000	Single	Mixed sludge	Batch mode	35	7.0	3	500	84	40	1700	4240	Nam et al. (2010)
2	84–10,000	Single	Mixed sludge	Continuous	35	6.5	40	3500	84	20	1.4	6.1	Kim et al. (2011)
3	6033	Single	Mixed sludge	Fed-batch	23	7.4	155	3000	900	50.3	1.6	344	Puig et al. (2011)
4	5000	Dual	Mixed sludge	Continuous	27	7	20	-	-	62	176	272	Rikame et al. (2012)
5	660	Dual	Anaerobic mixed sludge	Fed-batch	20	7.0	25	1500	200	85	1.67	2700	Zhang and He (2013)
6	100 to 4000	Single chamber	Mixed sludge	Fed-batch	23.2	7.1	6	4000	3000	80	1.7	2.0	Tice and Kim (2014)
7	908	Dual	Mixed sludge	Batch-continuous	24	6.5	52	200	430	90	653	824	Damiano et al. (2014)
8	3520.9	Double chamber	Mixed sludge	Batch-continuous	30	8.84	30	-	-	64.3	147.6	197.7	He et al. (2019)
9	3582	Single chamber	Pure culture	Continuous	35	7.18	35	300	1000	90	160	230	Li et al. (2017)
10	80–240	Single chamber	Mixed sludge	Batch-continuous	26	7	42	50	200	57	98.6	778	Hassan et al. (2018a, b)
11	503	Single	Mixed sludge	Fed-lab	22.3	8.00	50	800	64	60	32	60	Hiegemann et al. (2018)
12	500	Single chamber	Mixed sludge	Fed-batch	30	7	21	150	500	93	350		Li et al. (2019)
13	1000	Single chamber	Mixed sludge	Continuous	27	9	16	-	-	90	1.1	146	Feng et al. (2020)
14	3500	Double chamber	Pure culture	Continuous	35	7	11	-	-	76	267	348	Hai et al. (2020)
15	5000	Dual SC-MFC	Mixed culture	Continuous	35	6.9	18	1000	1500	78.2	178	298	Cai et al. (2020)
16	0.25–50	Single	Mixed sludge	Fed mode	30	7.1	5	0.25	50	50	299	373	Topçu et al. (2021)
17	29–2000	Combined single chambers	Mixed sludge	Fed-batch c	25	7.2	12	2000	500	63	0.8	1.2	Ergettie and Dagbasi (2021)
18	1–30	The single couple constructed a wetland	Mixed sludge	Batch-continuous	27	7.0	12	-	-	89.7	136	148.6	Wang et al. (2021)

*PD* power density, *Conc* concentration

### Some of the recent technological advances in leachate treatment and ammonia removal

Ammonia nitrogen ${(\mathrm{NH}}_{4}^{+}$-N) is the most common form of nitrogen in wastewater, and its release has become a global issue. Many strategies are employed to lessen ${\mathrm{NH}}_{4}^{+}$-N pollution in permitted water systems (Dong et al. 2019; Zhang et al. 2020). Isolating the landfill to prevent water or liquid from entering the landfill, returning leachate to the landfill, and treating leachate produced by the landfill are the three techniques of leachate management. Because leachate characteristics vary with season, landfill age, and other factors, such quantity and quality fluctuations must be considered while designing and operating a landfill leachate treatment system (El-Gohary and Kamel 2016; Zhang et al. 2020). This makes it difficult to meet the standards for landfill leachate discharge to a watercourse using only one treatment method, such as physical, chemical, or biological treatment. Several articles detailing feasible methods for treating landfill leachate have been published. There are certain limitations to these methods, though. Combining them has been shown to boost therapy efficacy in the past.

#### Biological techniques

Biological techniques can effectively remove COD, NH_3_-N, and heavy metals from young leachate (Renou et al. 2008). However, when the leachate contains excessive organic compounds, the activated sludge process may become clogged, increasing effluent concentration (Renou et al. 2008; Zhang et al. 2020). Pre-treatment is an alternate method for controlling the deficit by removing nitrogen from leachate and phosphorus from household wastewater. Aerobic, anaerobic, and anoxic biological activities are all in leachate (Hanira et al. 2017; Zhang et al. 2020). The removal of biodegradable chemicals from leachate is greatly facilitated by biological treatment. This approach may remove up to 50% of COD from clean landfill leachate (Gao et al. 2015; Zhang et al. 2020). However, the approach no longer applies to older landfills when the BOD/COD ratio is less than 0.1 (Gao et al. 2015).

#### Rainfall caused by chemicals

${\mathrm{NH}}_{4}^{+}$-N can be removed from wastewater using chemical precipitation. High ${(\mathrm{NH}}_{4}^{+}-\mathrm{N}.$ effluent can be separated using chemical precipitation by adding chemicals like magnesium chloride and phosphoric acid or phosphate. Tansel et al. (2018) and Huang et al. (2018) point to the importance of these factors in MAP’s evolution. Since ${\mathrm{NH}}_{4}^{+}$, Mg^2+^, and ${\mathrm{PO}}_{4}^{3-}$ does pH influence all? However, the significant expenditures and environmental damage caused by this treatment are mostly attributable to the massive quantities of chemicals used.

#### Constructed wetlands

Constructed wetlands CWs) are artificial ecosystems that filter out and reuse water from municipal, industrial, and agricultural sources (Wu et al. 2014). A constructed wetland is another viable biological option for leachate treatment. Plants, medium, and bacteria are all intentionally built into this system. Use water plants with long, stringy roots for the most effective leachate cleanup. Limnocharis flava, Ipomoaea Aquatica, and Scirpus validus can effectively remove ammonia nitrogen. Constructed wetland (CW) systems can successfully recover biodegradable organic carbon and ammonia from landfill leachate (Dan et al. 2017), as stated by Mojiri et al. (2016). There are several potential methods for reducing nitrogen pollution, which comprises adsorption onto substrates, uptake by plant roots, volatilization of ammonia, biological breakdown, and biochemical translation into N_2_ (Badejo et al. 2020; He et al. 2019).

#### Air stripping

Pre-treatment of wastewater using air stripping of ammonia is a common practise. The air stripping method is extensively employed in removing ammonia from wastewater, and large amounts of ammonia removal may be obtained with very cheap cost and simple equipment (Ozturk et al. 2003; Zhang et al. 2020). Therefore, air stripping is a good method for extracting and recovering valuable ammonia from wastewater because it provides a larger mass transfer surface; air stripping is frequently conducted in a packed tower to achieve high process efficiency (Zhang et al. 2020). Temperature, pH, stripping duration, and the ratio of air to liquid volume are all variables that influence stripping efficiency (Provolo et al. 2017), increasing expense and salinity, both of which harm biological treatment.

#### Membrane technology

A membrane is a thin, selective barrier that allows one component to flow freely while preventing another from doing so. Microfiltration, ultrafiltration, nanofiltration, and reserve osmosis are some membrane filtration technologies used in leachate treatment. However, membrane filtration methods cannot be used without pre-treatment or in combination with other membrane processes (Mojiri et al. 2013). There are different types of membrane technology, such as microfiltration, osmotic, nanofiltration, and ultrafiltration. These technologies can filter out potentially hazardous particles in the leachate.

#### Nitrification and denitrification

Nitrification and denitrification are processes that occur in the environment. The microbial removal of ammonium is involved in the denitrification and nitrification processes. During a typical nitrification–denitrification process, ammonia is converted into nitrate under aerobic conditions, which is then reduced to N_2_ under anoxic conditions (Zhang et al. 2020). The process begins with ammonia being converted to nitrite (${\mathrm{NO}2}^{-}$) by ammonia-oxidizing bacteria. Second, bacteria that convert ${\mathrm{NO}2}^{-}$ to nitrate are known as nitrite-oxidizing bacteria. Finally, heterotrophic bacteria engage in nitrate denitrification to generate N_2_ during the anoxic phase because of other contaminants’ impact on the process (Miao et al. 2019). This phase is usually included with other treatment methods. Tałałaj et al. (2016) employed a suspended-carrier biofilm reactor to examine the potential nitrification of leachate and discovered that temperature had only a minor influence, but HRT had a significant impact. Zhang et al. (2020) achieved 85 to 90% nitrogen removal at an NH_4_^+^-N loading rate of 1.2 g m^−3^ day^−1^ and HRT of 1 day.

#### Co-treatment of landfill leachate with domestic wastewater

To lower the content of high organic components like ammonia, leachate wastewater is diluted with domestic wastewater. To improve biodegradability and to balance BOD/COD ratio in landfill leachate treatment, researchers mixed domestic wastewater with landfill leachate wastewater before treatment (Mojiri et al. 2016). However, most of the methods had one or more drawbacks; until recently, bioelectrochemical systems (BES) received significant attention as they enable the use of microorganisms as promoting agents or catalysts to convert the chemical energy of the electron donors into electricity.

#### Ion exchange and adsorption

Ion exchange removes ammonia. Many ion exchangers and adsorbents, including zeolite, have been used for years (Huang et al. 2018). Due to its strong ion exchange capacity and distinctive pore structure, zeolite is now the most widely utilized ion exchanger. pH, temperature, particle size, starting ammonium concentration, contact duration, and adsorbent dose all affect ${\mathrm{NH}}_{4}^{+}-\mathrm{N}$ adsorption. The pH of the solution has a big impact on ammonia adsorption (Dong et al. 2014). If the pH is > 7.0, the ${\mathrm{NH}}_{4}^{+}$ is transformed to NH_3_, which cannot exchange onto the adsorbent. At pH 5.0, H^+^ competes with ${\mathrm{NH}}_{4}^{+}$ for adsorption sites, decreasing ${\mathrm{NH}}_{4}^{+}$ removal. The optimal pH has been reported to range from 5 to 8 (Huang et al. 2018). Natural zeolites were used to remove percent ammonium under ideal conditions: pH 5, temperature of 25 °C, contact period of 8 h, starting ammonium content of 50 mg L^−1^, and zeolite loading of 1 g per 100 mL (Deng et al. 2021).

#### Breakpoint chlorination

Breakpoint chlorination is the process of adding an excessive amount of chlorine or sodium hypochlorite to wastewater to convert ${\mathrm{NH}}_{4}^{+}$-N to N_2_, which is subsequently discharged into the atmosphere. When Cl_2_ is added to wastewater, the free chlorine content decreases to its lowest value, which contains very little ammonia (Dong et al. 2019). When extra Cl_2_ is supplied indefinitely, the concentration of free chlorine increases, and a breakpoint is formed. This approach is frequently used as an advanced treatment; however, it is not suitable for treating large amounts of wastewater with high ${\mathrm{NH}}_{4}^{+}$-N levels. Zhang et al. (2020) showed that combining UV irradiation at 254 nm with chlorination increased the ammonia removal rate and efficiency compared to breakpoint chlorination alone.

### Bio electrochemical systems

Microorganisms in a bioelectrochemical system (BES) transform the chemical energy stored in biodegradable materials into electric current and chemicals. As a versatile platform for oxidation and reduction reaction-oriented processes, BES provides a novel option for integrated waste treatment and energy and resource recovery (Logan and Rabaey 2012). Most BES reactors feature an anode, a cathode, and a separator (though this last component is not required), with several configurations available for different purposes. In a microbial fuel cell (MFC), microorganisms oxidize organic matter, such as wastewater, in the anode chamber to generate electron flow (current) to the cathode, where the electrons can be used for direct electricity production or the reduction of water or oxidized chemicals, such as metal ions, carbon dioxide (CO_2_), or organic chemicals (in a microbial electrolysis cell (MEC) or microbial electrosynthesis (MES).

The effectiveness of BES in treating wastewater has been studied extensively in recent years, and its applicability to various wastewater conditions has been the subject of several outstanding studies (Pant et al. 2010). Research has consistently found that BES improves wastewater treatment by reducing costs associated with aeration energy and sludge disposal. BES has been predominantly employed for weaker wastewater treatment (Zhang and He 2013) due to its lower energy densities than other anaerobic processes, such as anaerobic digestion.

BESs have recently emerged as a potentially useful and difficult method of producing bioelectricity. Despite the quick advancement, more study is needed to overcome the limitations of BES in several domains. Despite having many useful features and a wide range of potential applications, BESs have mostly stayed in lab-scale or demonstration projects. Several factors have hampered bioelectrochemical technology efforts to scale up, but low efficiency and a slow pace of production stand out as the most significant (Zhang et al. 2016). There is a clear information gap in calculating the efficacy of BESs on wastewater treatment, as most BES publications are focused on the electricity generation component. Previous research has shown that the MFC system is highly effective, removing many organic and nutrient substrates from various wastewater, including leachate wastewater.

#### Mechanisms of BES

Mohanakrishna et al. (2015) found that bioenergy might be produced via microbial (catabolism or anabolisms) reactions in BFCs. This occurs due to a synthesis of oxidative (fermentation, putrefaction) and anaerobic (respiration, reduction) reactions. At the anode, oxidation reactions such as fermentation and putrefaction typically occur (Kim et al. 2007). When this happens, an external circuit connects it to a cathode electrode so that electrons can flow between them. Consequently, the fuel cell can harness the energy produced by bacterial respiration. However, a biocatalyst, such as bacteria or yeast, is required for these processes to work. The substrate or fuel is broken down by the biocatalysts into electrons, protons, and an active/weak electron acceptor (i.e., an anode, often graphite) to finish the cyclic movement of electrons (Zhang et al. 2020) by isolating fermentation from respiration using a PEM in a system with artificial electron acceptors, an optimal environment is created for growing energy in the form of current density against the potential difference (PD) between these two processes.

#### MFC principles

The standard MFC configuration includes the following parts: anode chamber, cathode chamber, separator, and external circuit. Microorganisms in the anode solution oxidize organic pollutants, releasing electrons and protons, which are then transferred to the cathode through an external circuit and energy, are released to supply the electron acceptor (Muaz et al. 2019; Wang et al. 2019). Ultimately, the MFC’s cathode electrons and protons react with oxygen, creating a reduction reaction that yields water molecules to create a circuit and generate an electric current. The microbe may obtain more energy, and therefore, more power can be generated by MFC if the potential difference between the electron donor and the electron acceptor is larger (Talukder et al. 2017; Zhang et al. 2020).

#### Types of electrodes

An electrode is a tiny piece of metal or other material or substance used to carry electricity in contact with a non-metallic component of a circuit. It might be a biological organism or a piece of machinery that could be used to create a circuit. There are two main types of electrode materials, the anode and the cathode. Good conductivity, enhanced mass transfer, and a broad surface area with conveniently accessible holes are all considered when choosing an electrode for a biofuel cell. The materials are also biocompatible, chemically stable, scalable, and have a cheap cost (Zhang et al. 2020). There are two primary ways in which the material used to create the electrodes will impact their electrical performance: The electrode’s surface roughness comes first. Microorganisms’ ability to adhere to an electrode surface is affected by how smooth the electrode surface is. According to research, more biomass means better production efficiency (Kumari et al. 2018). This is because the closer the electrode surface roughness and the microbial cell size, the stronger the microbial adhesion ability. To continue, we have electrode material modification. Since graphene and carbon nanotubes have high electrical conductivity and catalytic activity, they are widely considered to be a promising area for future study.

#### MFC configurations

Single-chamber, dual-chamber, and stacked MFCs are common MFCs utilized in research. Single-chamber MFCs have a single reaction chamber that serves as the cathode and anode. Without additional ventilation, the cathode is open to the air, resulting in a low resistance, a high electron transmission rate, and no secondary pollutants. It is possible to tailor the setup of a microbial fuel cell (MFC) to meet specific performance requirements. A typical MFC has two chambers, or halves, with the anode and cathode electrodes separated by a membrane to prevent microbial growth on the cathode. Substrates and microorganisms are placed in the anode chamber to oxidize the organic molecules and transfer electrons to the anode electrode. To mix with oxygen or another electron acceptor, electrons go over an external circuit to the cathode electrode (Zhang et al. 2020). The anode and cathode electrodes of a single-chambered system and stacked MFCs, in which numerous MFCs are stacked together to improve power output, are two more MFC configurations. When designing and operating an MFC, keeping the configuration in mind is important because it will significantly impact the device’s performance (Zhang et al. 2020). Still, more research is needed to clarify the impedance effect of stacked MFC.

#### Types of separation materials (membrane)

Keyikoglu et al. (2021) explain that the primary distinction between dual-chamber and single-chamber MFCs is the presence or lack of a separation material. The separation material greatly affects the transfer rate and electrical performance, as the hydrogen protons created by peroxidation in the anode chamber must be transferred to the cathode chamber. The spacer material boosted proton migration to accommodate the differences between cathode and anode in substrate composition, dissolved oxygen concentration, microbial kind, etc.

Proton exchange membranes (PEMs), cation exchange membranes (CEMs), and anion exchange membranes (AEMs) are only some of the membrane materials used in MFCs. Physical and chemical characteristics, as well as selective transmittance, vary greatly between membrane materials. In addition to successfully decomposing pollutants, membrane bioreactor technology has been cited in the literature for its ability to retain biomass (Ahmed and Lan 2012). Microalgae are also considered a promising source of raw materials for the biofuel industry (Wu et al. 2015; Zhang et al. 2020).

#### Anodic microorganism

Forms of microorganisms generating electricity. The species and activity of bacteria on the surface of MFC are crucial to enhance the electrical performance of MFC since they can decompose organic materials while also generating energy at the anode. Proteobacteria, Escherichia coli (Zhang et al. 2020), Geobacter, Helicobacter (Richter et al. 2008), Firmicutes, Acidobacteria, and Actinomycetes are only a few of the microbial groups that have been detected in the anode and are capable of creating electricity. The most common microorganisms employed in studies of MFCs are Shewanella and Geobacter. In recent years, solid metal oxide–reducing bacteria have been the primary source of high-efficiency electricity-producing microorganisms. They are Gram-negative proteobacteria because the MFC’s operating environment is comparable to bacterial habitats and uses the same electron transfer process. It was discovered by Dalun et al. (2021) that the types of bacteria found on the surface of the electric-producing anode would change greatly depending on the substrate. However, it was found that several types of bacteria appeared in them. These bacteria included Gram-positive bacteria with low G + C, − Proteobacteria, and − Pro-mycobacteria, which may be related to auxiliary bacteria that produce electricity.

#### Mixed bacteria MFC

Compared to MFC made from pure bacteria, MFC made from mixed bacteria has a greater resistance to environmental changes, making it easier to alter the experimental conditions to study a single factor in greater depth benefiting from the synergistic effect of the bacteria. Potential future MFC production benefits greatly from this finding. Compared to pure MFC, mixed microbial flora MFC generates around six times as much energy due to an enrichment of the dominating flora on the anode surface (Albarracin-Arias et al. 2021). However, some literature also pointed out that electric-producing bacteria and non-electric-producing bacteria had a competitive relationship in the degradation process of organic matter, so the electric-producing performance of mixed bacteria MFC was lower than that of pure bacteria MFC (Lu et al. 2021).

#### Factors affecting the electrical performance of MFC

Researchers worldwide have been looking into MFC to produce renewable electricity while also cleaning up wastewater in recent years. However, there is a dearth of literature on optimizing MFC’s investment cost in relation to its electricity output and designing more cost-effective and efficient systems (Zhang et al. 2020). Reactor structure, substrate composition, pH, temperature, operating conditions, electrode material, microbial community, and so on are all examined as potential influences on MFC electrical performance.

- Substrate type and concentration: The substrate type and concentration used in the MFC can significantly impact its electrical performance. Some substrates are more easily metabolized by the microorganisms, resulting in higher power output, while others may be less efficient or even inhibitory.
- Microbial community: The composition of the microbial community in the MFC can affect its electrical performance. Some microorganisms are better suited for electron transfer and electricity generation, while others may compete for resources and reduce overall performance (Haslina et al. 2021; Zhang et al. 2020).
- Electrode material: The electrical performance of the MFC might be affected by the material used for the electrodes. Their unique features can affect the creation of current and the movement of electrons between materials.
- pH and temperature: The MFC’s pH and temperature can affect the microorganisms’ activity and ability to transfer electrons. Optimal conditions for microbial growth and metabolism may differ from optimal conditions for electricity generation, so balancing these factors is important (Kim et al. 2007; Zhang et al. 2020).
- Electron acceptors: MFCs require electron acceptors to maintain the flow of electrons and generate electricity. Different types of electron acceptors can have varying effects on electrical performance.
- Toxic compounds: Toxic compounds, such as heavy metals or organic pollutants, can inhibit microbial growth and metabolism and reduce the electrical performance of the MFC.
- Operating conditions: The operating conditions of the MFC, such as flow rate, reactor design, and electrode spacing, can also affect its electrical performance.

### Mechanism of ammonia inhibition in MFC operation

The significance of comprehending and resolving ammonia inhibition in MFCs is reviewed in this article. Agricultural runoff, home sewage, and industrial wastewater are common pollutant ammonia nitrogen (NH_3_-N) sources.

The microbial mechanism of ammonia inhibition in the microbial fuel cell (MFC) system can be attributed to the detrimental effects of high ammonia concentrations on the activity of the microorganisms involved. Ammonia can have toxic effects on microbial metabolism and disrupt the electron transfer processes essential for electricity generation in MFCs (Dai et al. 2020). When ammonia is present in the MFC system, it can penetrate the microbial cell membranes and interfere with the microorganisms’ metabolic pathways and enzymatic reactions. Ammonia can inhibit the activity of key enzymes involved in organic matter oxidation, electron transfer, and energy generation, thereby reducing the MFC’s overall microbial activity and power output (Lu et al. 2021). Additionally, ammonia can disrupt the proton gradient across the cell membrane, which is crucial for electron transport and ATP synthesis. As a result, high ammonia concentrations can impede the overall microbial performance and compromise the efficiency of the MFC system (Deng et al. 2021).

Excessive levels of ammonia compromise the efficiency of leachate treatment in addition to causing health and environmental issues (Haslina et al. 2021). However, microbial fuel cells (MFCs) use microorganisms’ catalytic activity to turn organic material into power. Ammonia is one of the most frequent inhibitory chemicals in MFCs since it can interfere with microbial metabolism and lower system efficiency. When dissolved in the aqueous phase, ammonium (${\mathrm{NH}}_{4}^{+}$) predominates; the production of NH_3_ is regulated by TAN concentration, pH, and temperature. The NH_3_ content is thought to be the active ingredient that suppresses biological activity. According to earlier research (Wang et al. 2021), for unadopted microbial cultures, the formation of ${\mathrm{NH}}_{4}^{+}$ ions inside a cell might cause inhibition by altering intracellular pH conditions. Unionized NH_3_ can directly block cellular enzyme performance. Because of the long-term recirculation caused by the presence of NH_3_-N in MFC, other bacterial groups were inhibited, and some microorganisms, like Iodidimonas, were able to play an important part in the inhibition (Li et al. 2020). The literature claims that various operational and design variables, such as pH and electrical conductivity, impact MFC performance (Kim et al. 2007; Logan et al. 2006). Furthermore, high ammonia concentration may cause bacterial dehydration due to osmotic stress and act as a building component for the microorganism’s transport route to the anode electrode (Chen et al. 2018). NH_3_-N can reduce the power output of MFCs by inhibiting the activity of electrochemically active microorganisms (EAMs) that are responsible for generating electrical currents. This inhibition can occur due to the toxicity of NH_3_-N to EAMs, which can cause cell damage and reduce metabolic activity. As a result, the performance of MFCs can be significantly reduced, and the power output can drop by up to 50% in some cases. (Liu et al. 2021). NH_3_-N can also accumulate in MFCs and be converted to ammonium, which can further inhibit the activity of EAMs. Ammonium can also cause pH changes in the MFC, affecting the activity of microorganisms and reducing the performance of MFCs (Ganjian et al. 2018).

#### Principles of ammonia–nitrogen formations:

Ammonia nitrogen (TAN) can be formed in microbial fuel cells (MFCs) through several processes. One major source of TAN in MFCs is the breakdown of nitrogen-containing compounds in the wastewater or other feedstock used as a substrate. Microorganisms in the MFC, such as bacteria and archaea, utilize enzymes like proteases to break down complex organic compounds, including proteins and amino acids.

Therefore, ammonia is produced as a waste product during decomposition. The formed ammonia can then react with the solution’s water to produce ammonium ions, ${\mathrm{NH}}_{4}^{+}$, which are the most common type of nitrogen in solutions. Under the combined activity of microorganisms, biological nitrogen removal consists of organic nitrogen ammonification, nitrification, denitrification, and microbial absorption (Shou et al. 2019). Among the organic N molecules in wastewater, proteins, amino acids, urea, amine, and nitro compounds predominate. Deamination refers to the breakdown and transformation of organic nitrogen compounds by ammonifying bacteria, followed by ammonia release under aerobic or anoxic circumstances (Li et al. 2021). Nitrification is the aerobic oxidation of ammonia nitrogen ${\mathrm{NH}}_{4}^{+}$ by ammonia-oxidizing bacteria and nitrite-oxidizing bacteria to form nitrite (${\mathrm{NH}}_{4}^{+}$-N) to ${\mathrm{NO}}_{2}^{-}$-N and ${\mathrm{NO}}_{3}^{-}$-N, respectively. Denitrification is the process by which bacteria use organic carbon as electron donors and reduce to gaseous nitrides in anaerobic or anoxic environments using NO_3_^−^ or NO_2_^−^ created during the nitrification process as electron acceptors (Huang et al. 2018).

The overall reaction for this mechanism can be represented as:

i. Ammonification: Organic nitrogen compounds (e.g., urea) + microorganisms → ammonium ions (NH_4_^+^). This reaction can be described by the following chemical equation:
  1 $${\mathrm{CH}}_4{\mathrm N}_2\mathrm O+{\mathrm H}_2\mathrm O+\mathrm{bacteria}\rightarrow\mathrm{NH}_4^++{\mathrm{CO}}_2+{\mathrm{NH}}_3$$

In this reaction, urea (CH_4_N_2_O) is a common organic nitrogen compound present in landfill leachate, and bacteria convert it into ammonium ions (${\mathrm{NH}}_{4}^{+}$) and ammonia (NH_3_) through the process of ammonification. The reaction also generates carbon dioxide (CO_2_).

ii. Nitrification:
  2 $$\mathrm{Ammonium}\;\mathrm{ions}\;(\mathrm{NH}_4^+)+\mathrm{oxygen}+\mathrm{nitrifying}\;\mathrm{bacteria}\rightarrow\mathrm{nitrite}\;\mathrm{ions}\;\left(\mathrm{NO}_2^-\right)+{\mathrm H}_2\mathrm O$$
  3 $$\mathrm{NH}_4^++1/2\;{\mathrm O}_2+\mathrm{bacteria}\rightarrow\mathrm{NO}_2^-+{\mathrm H}_2\mathrm O,$$
  4 $$\mathrm{Nitrite}\;\mathrm{ions}\;\left(\mathrm{NO}_2^-\right)+\mathrm{oxygen}+\mathrm{nitrifying}\;\mathrm{bacteria}\rightarrow\mathrm{nitrate}\;\mathrm{ions}\;\left(\mathrm{NO}_3^-\right)+\mathrm{water},\;\mathrm{NO}_2^-+1/2\;{\mathrm O}_2+\mathrm{bacteria}\rightarrow\mathrm{NO}_3^-+{\mathrm H}_2\mathrm O$$

iii. De-nitrification:
  5 $$\begin{array}{c}\mathrm{Nitrate}\;\mathrm{ions}\;\left(\mathrm{NO}_3^-\right)+\mathrm{anaerobic}\;\mathrm{bacteria}\rightarrow\mathrm{nitrite}\;\mathrm{ions}\;\left(\mathrm{NO}_2^-\right)+\mathrm{nitrogen}\;\mathrm{gas}\;\left({\mathrm N}_2\right)+{\mathrm H}_2\mathrm O\\\end{array}$$
  6 $$\mathrm{NO}_3^-+\mathrm{bacteria}\rightarrow{\mathrm{NO}}_{2-}+{\mathrm N}_2+{\mathrm H}_2\mathrm O,\mathrm{Nitrite}\;\mathrm{ions}\left({\mathrm{No}}_{2-}\right)+\mathrm{anaerobic}\;\mathrm{bacteria}\rightarrow\mathrm{nitrogen}\;\mathrm{gas}\;\left({\mathrm N}_2\right)+\mathrm{water}\;\mathrm{NO}_2^-+\mathrm{bacteria}\rightarrow{\mathrm N}_2+{\mathrm H}_2\mathrm O$$

The ammonia nitrogen formed through these mechanisms can benefit and harm MFC performance. On the one hand, ammonia nitrogen can serve as a nitrogen source for microorganisms and enhance their growth and activity.

#### The impact of ammonia inhibition on various variables in MFC operation

Ammonia in wastewater can greatly impact how well microbial fuel cells work (MFCs). It functions as an electron acceptor and competes with the anode for electrons, which can prevent electrochemically active bacteria from growing and operating. This is necessary for an MFC to operate properly. A few MFC operation characteristics that could be influenced by ammonia inhibition include power output, current density, and COD removal efficiency.

Energy produced: The electricity production by MFCs is decreased by ammonia inhibition because it restricts the flow of electrons from the anode to the cathode. To decrease the amount of current, the MFC produces this is done. Nitrogen ammonia can steal electrons from the anode and interfere with the flow of electrons to the cathode. The system’s voltage and power output are thus decreased.

i. Current density: Since ammonia nitrogen inhibits the electrochemical activity of bacteria that transport electrons to the anode, it can also reduce the current density of MFCs. Due to a drop in electron transfer due to a decrease in electrochemically active bacterial activity, current density drops (Damiano et al. 2014).
ii. COD removal efficiency: MFCs can remove organic materials from wastewater while generating power, so they are frequently utilized. Ammonia inhibition reduces the activity of the microorganisms responsible for organic matter decomposition, lowering the COD removal efficiency of MFCs. Another way in which ammonia nitrogen might inhibit the microbes responsible for organic matter breakdown is by altering the system's pH (Huang et al. 2018).
iii. pH: Nitrogen ammonia can also lower the pH of MFCs. Because of its weak basic character, ammonia can elevate the system’s pH. The microorganisms responsible for the breakdown of organic materials and their growth could be stunted. Precipitation of specific salts in the system due to an alkaline pH can also lead to scaling and clogs (Gutierrez et al. 2016).
iv. Temperature: How much of an impact ammonia has on MFC temperatures depends on how much nitrogen is present in the ammonia. The ammonia nitrogen can produce heat in large quantities, raising the system’s temperature. The MFC’s power output and COD removal efficiency may suffer if the temperature rises above a certain threshold (Cheng et al. 2011).
v. Electric conductivity: Because it controls how easily electrons may move through the system, electric conductivity is crucial in MFCs. The presence of ammonia in the MFC can cause disruptions in electron transport by binding to the bacterial cell’s membrane. Reduced power output from the MFC is a possible consequence of this phenomenon (Hirata and Ohtaki 2020). To keep the MFC operating at peak performance, ammonia levels must be tightly regulated to ensure consistent electrical conductivity (Lee and Joo 2021). Since NH3-N’s involvement in MFCs is complex and relies on several parameters, understanding its impact is essential for enhancing their performance and developing sustainable leachate treatment and renewable energy generation methods. This article reviews the current knowledge about the inhibition of MFCs by ammonia. Previous research has described the effects of different ammonia concentrations on MFCs, as shown in Table 3.

Table 3 summarizes studies on the performance of microbial fuel cells (MFCs) in removing nitrogen-containing compounds from landfill leachate. The table reports influent concentrations of ammonia–nitrogen compounds, reactor types, inoculation, modes, temperature, pH, period, inhibiting, optimum concentrations, removal efficiency, minimum and optimum power densities, and conclusion from different studies.

Previous research summarized in Table 3 examined the relationship between influent substrate concentration and ammonia inhibition in single-chamber MFCs and double-chamber MFCs. It may be concluded that high power densities and a greater enrichment of anammox bacteria for efficient treatment and power density potential were caused by high ammonia–nitrogen influent concentrations. Studies have shown that maintaining a high substrate concentration in MFCs with high concentrations of TAN can lead to substantial current generation (Ali et al. 2020; Wang et al. 2021) (Table 3). A high substrate concentration can provide the necessary nutrients for the microorganisms to thrive, even with high ammonia levels. However, poor substrate conditions can significantly reduce current generation in MFCs, even under low concentrations of TAN. This suggests a specific substrate concentration should be offered to maintain active exoelectrogenic bacteria under high ammonia conditions. The reactor types used in the studies include single and double chambers, single supercapacitor chambers, and dual SC-MFC. The size of the MFC reactor can affect the concentration of ammonia nitrogen in several ways. A larger reactor can accommodate a larger volume of wastewater containing ammonia nitrogen, which can increase the overall concentration of ammonia nitrogen in the system.

However, a larger reactor can also provide more surface area for biofilm formation and microbial growth, which can help to mitigate the negative effects of ammonia nitrogen by providing a larger population of microorganisms that can metabolize the ammonia (Wang et al. 2017). This clearly describes Table 3 as most MFCs with double chambers and high working volume conditions exhibit high performance, which could effectively convert ammonia–nitrogen in the anode chamber. However, The optimal reactor size will depend on the specific operating conditions, including the concentration of ammonia nitrogen in the wastewater, the type of microorganisms present in the system, and the desired performance outcomes. Most studies employed a mixed sludge inoculation mode, with some using a pure culture inoculation mode. Table 3 describes mixed culture as widely used for injection because mixed culture sludge inoculation typically involves a complex microbial community that contains a variety of microorganisms, including bacteria, archaea, and fungi. These microorganisms can degrade various organic compounds, including ammonia nitrogen. Ammonia nitrogen is an important parameter in anaerobic digestion, as it can inhibit the activity of microorganisms if present in high concentrations. Mixed culture sludge injection has improved the anaerobic digestion in high ammonia nitrogen levels. This is because mixed cultures are more robust and can adapt to changes in environmental conditions, such as high levels of ammonia nitrogen (see Table 3). This confirms the previous studies (Hassan et al. 2019). While pure culture injection can lead to a more predictable and controlled microbial community, it may not be as efficient at degrading complex organic matter as mixed culture inoculation. This can be observed in Table 3, as most of the studies critically utilized mixed culture because of the diversity and complexity of the microorganisms (Ilmasari et al. 2022). The presence of a single strain of microorganisms may make the MFC more vulnerable to the inhibitory effects of ammonia nitrogen. The exoelectrogenic microorganisms could generate current at a maximum capacity (3.8 mA) for all MFC reactors at TAN concentrations up to 2500 mg L^−1^ (Tice and Kim 2014).

The studies used different temperature and pH ranges, but the temperature range was mostly between 20 and 35 °C, while the pH range was mostly between 6.5 and 8.84. At high concentrations, ammonia nitrogen can cause a decrease in pH due to the production of acidic by-products during microbial metabolism. However, most studies that reported high energy output and treatment are within a neutral pH range (7–7.5) (Table 3). Neutral pH resulted in a power shift and reduced inhibition’s effect in MFCs. The production of acidic and alkaline by-products during microbial metabolism of ammonia can be minimized, reducing the inhibition of microbial activity and maintaining optimal MFC performance.

Studies have shown that increasing the MFC’s operating temperature can help mitigate the negative effects of ammonia inhibition. At higher temperatures, the rate of microbial metabolism of ammonia nitrogen can increase, leading to higher power output and better MFC performance. However, if the temperature becomes too high, it can cause denaturation of the microbial enzymes and inhibition of their activity, leading to decreased MFC performance. Most of the studies were done at room temperature (25–30 °C), which could result from difficulties controlling the MFCs at higher temperatures, and some of the microorganisms hardly survive in higher temperatures. The increased metabolic activity can also help to overcome the inhibitory effects of high ammonia concentrations. However, it is important to note that there is an upper limit to the temperature that can be used in MFCs, as temperatures that are too high can cause denaturation of the microbial enzymes and inhibition of their activity (Nor et al. 2015). Temperature can play an important role in suppressing ammonia inhibition and methane suppression in MFCs.

The periods of the studies reported in Table 3 ranged from 3 to 52 days. The longer the MFC operates, the higher the likelihood of experiencing ammonia inhibition. This is because as the microorganisms in the MFC consume organic matter in the wastewater, the concentration of ammonia nitrogen can increase, which can lead to the inhibition of microbial activity. Additionally, the accumulation of microbial biofilms in the MFC can lead to increased ammonia adsorption and accumulation, further exacerbating the problem.

The inhibiting concentration of TAN or ammonia nitrogen and the optimum concentration for power generation varied among the studies. Studies have shown that higher substrate concentrations can increase power output in microbial fuel cells (MFCs), as in Table 3 However, the optimum substrate concentration for maximum power output can vary depending on the specific study and experimental conditions. For example, some studies have reported an optimum substrate concentration range of 50 to 3000 mg/L, while others have found different optimal ranges. The inhibitory effect of higher substrate concentrations on MFC performance is likely due to factors such as increased internal resistance and substrate inhibition of the microorganisms (Xiang et al. 2020).

The minimum power density (PD) recorded was 1.1 mW/m^3^, while the maximum was 272 mW/m^3^. The removal efficiency ranged from 62 to 90%. Most of the studies in Table 3 reported that the total ammonia nitrogen (TAN) inhibiting concentration MFC was greater than 500 mg L^−1^ (Kim et al. 2011; Li et al. 2021). However, this can be affected by several factors. In a batch MFC, a high TAN concentration of > 500 mg L^−1^ may significantly stifle power production and impede efficient substrate removal (Joo et al. 2015). Similarly, Kuntke et al. (2018) found that ammonia at concentrations as high as 4000 mg L^−1^ had no negative effects. Thus, this review paper aims to explain the observed discrepancy in the TAN concentrations required to inhibit ammonia in MFCs. However, the present understanding of ammonium monitoring in leachate is grossly inadequate (Yang et al. 2021). according to Nam et al. (2010). A range of substrates, operational settings, biological variables, and the MFC setup system may all influence ammonium thresholds.

The maximum and minimum inhibition caused by TAN in MFC has been discussed by many. However, many research questions must be addressed, especially the optimum Inhibiting ratios in response to operating variables. Ammonia nitrogen inhibition remains a significant challenge for the optimal performance of MFCs. Despite numerous studies conducted to understand the mechanism of ammonia inhibition, more research is needed to improve the performance of MFCs.

#### Contributions and limitations of the current review

Bibliometric analysis is useful for investigating the interrelations between published articles, giving researchers a fresh viewpoint on a certain research topic. This study can highlight crucial research trends and patterns while also allowing us to watch the advancement of a certain field in a holistic approach. In MFC and landfill leachate research, key phrase analysis indicated two important study topics and the most productive authors and referenced articles were evaluated, allowing researchers to stay on top of field advances. Furthermore, the growth trends, classifications, and journals linked with MFC and landfill leachate investigations have been detailed, laying the groundwork for future research. The study provides a useful tool for policymakers, funding agencies, and other stakeholders to assess the impact of research in this field and allocate resources accordingly. The study reveals the most influential publications, helping researchers to identify the most important research studies and the significant findings in this area.

Nonetheless, it is crucial to remember that the data sources employed may limit the findings of bibliometric analysis. While the analysis searched the Web of Science and Scopus platforms for terms related to MFC and landfill leachate, some papers may have been excluded due to using alternative terminology or not being indexed in the database. As a result, researchers should interpret the data cautiously and employ additional methodologies to guarantee a thorough grasp of the research landscape. The analysis is based on quantitative measures, such as citation counts, which may not always accurately reflect the quality or relevance of research studies. Language barriers may limit the analysis, as research studies published in languages other than the language of analysis may be excluded. When merging multiple items in Zotero, the metadata from the two items is combined. However, the merged item may not contain all the relevant metadata from both items, which can lead to incomplete or inaccurate bibliometric analysis.

### Summary of findings and future recommendations

The collection includes 98 items, such as books and periodicals, from 2006 to 2023. According to the numbers, the median age of the documents is 5.35 years, and the number of publications is increasing at a pace of 10% each year. This would indicate that the publication rate has not changed significantly over time. With an average of 21.88 citations per document, it is clear that these pieces of work are well-referenced and will make substantial contributions to their respective fields of study. The dataset also includes 4060 references, demonstrating the authors’ thoroughness and the breadth of their study and citations. The dataset has 343 keywords plus 274 author keywords, indicating its breadth of coverage. This raises the possibility that the documents pertain to various disciplines. There are 362 writers spread over the documents, with just a single-author submission. This suggests that collaboration is common in this field. The dataset consists of 88 articles and 10 proceedings papers. This shows that most of the documents are research articles published in journals. The co-authorship rate is 5.26, which suggests a high level of collaboration among the authors. Additionally, 21.43% of co-authorships are international, indicating that the authors have a global perspective on their research.

- Using alternative anode materials, such as graphite felt, carbon cloth, and modified carbon materials, has increased the MFC's tolerance to high ammonia nitrogen concentrations. This aligns with the future research trend of ammonia nitrogen inhibition in MFCs, which involves investigating ways to mitigate the inhibitory effect of high ammonia nitrogen concentrations on MFC performance (Liu et al. 2021; Shou et al. 2019).
- Finding and describing the microbial communities that can efficiently transform high ammonia nitrogen concentrations into safe by-products, reducing the negative impacts of high ammonia nitrogen concentrations on MFC performance (Long et al. 2019). This can be done by conducting tests to determine the impact of environmental conditions, such as pH, temperature, and substrate type, on the abundance and activity of ammonia-oxidizing bacteria and archaea and metagenomic and metatranscriptomic analysis.
- The investigation of novel, ammonia-inhibition-resistant microbial strains. Increasing the microbial community’s capacity to withstand high ammonia levels may also require the application of bioaugmentation or genetic engineering.
- To lessen ammonia nitrogen inhibition in MFCs, reverse electrodialysis (RED), built wetlands (CWs), and membrane bioreactors (MBRs) can all be utilized in conjunction with MFCs (Zhang et al. 2020; An et al. 2021). This can increase the MFC’s ability to generate power while improving the removal efficiency of ammonia nitrogen.
- To broaden the variety of MFC applications in wastewater treatment and resource recovery, future research should create novel methods for reducing the inhibitory effects of high ammonia nitrogen concentrations on MFC performance.
- Researchers should use caution when interpreting the results of bibliometric analysis and consider the entire research landscape.

## Conclusion

The current research conducted a bibliometric analysis to evaluate the technological progress of chosen treatment methods quantitatively. This form of analysis can aid in comprehending the many facets of a particular research subject. The findings revealed that the performance of MFCs is hindered by varying concentrations of TAN. The inhibiting concentration range reported by previous studies is between 1000 and 1500 mg L^−1^, and the total average percentage removal was above 50%, as described in Table 3. Most research on treating landfill leachate pollutants by MFC technology has concentrated solely on a general treatment of the pollutants without considering the effect of optimal ratios of TAN inhibition. The latter is known to have a significant inhibitory influence on methanogenic activity, resulting in decreased performance and instability. Although, little research has studied the behaviour of microbial diversity and community structure of these microorganisms and their effects on electrochemical performance and treatment efficiency. More research is needed to optimize the performance and efficiency of MFCs in treating landfill leachate.

## Data Availability

The data used in the manuscripts are included in the text.
